# Supplementary material for: Interaction between Fish Skin Gelatin and Pea Protein at Air-Water Interface after Ultrasound Treatment
Source: Foods. 2022 Feb 23;11(5):659. doi: 10.3390/foods11050659 (PMC8909765; doi:10.3390/foods11050659)
Supplement: Supplementary file 1 [file foods-11-00659-s001.zip › foods-1579939-supplementary.pdf]

Supplementary Materials

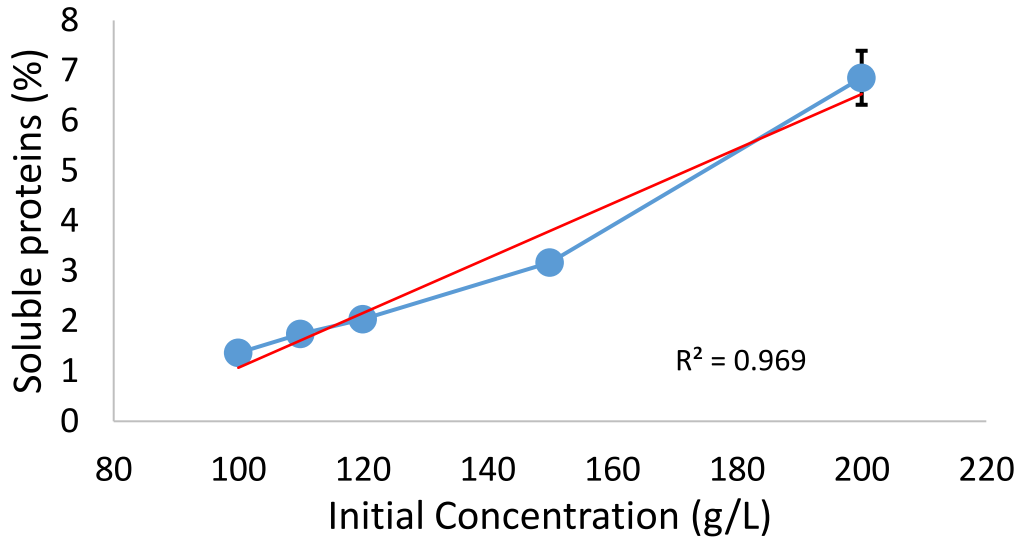

Figure S1. Calibration line of PPI supernatant soluble protein content (%). 5 different starting concentrations were analysed by Dumas method after the removal of the insoluble part.

Table S1. Soluble protein content of different starting solution concentrations.

C (g/L)	Soluble Protein (%)
100	1.4 ± 0.03
110	1.8 ± 0.02
120	2.1 ± 0.01
150	3.2 ± 0.03
200	6.9 ± 0.54

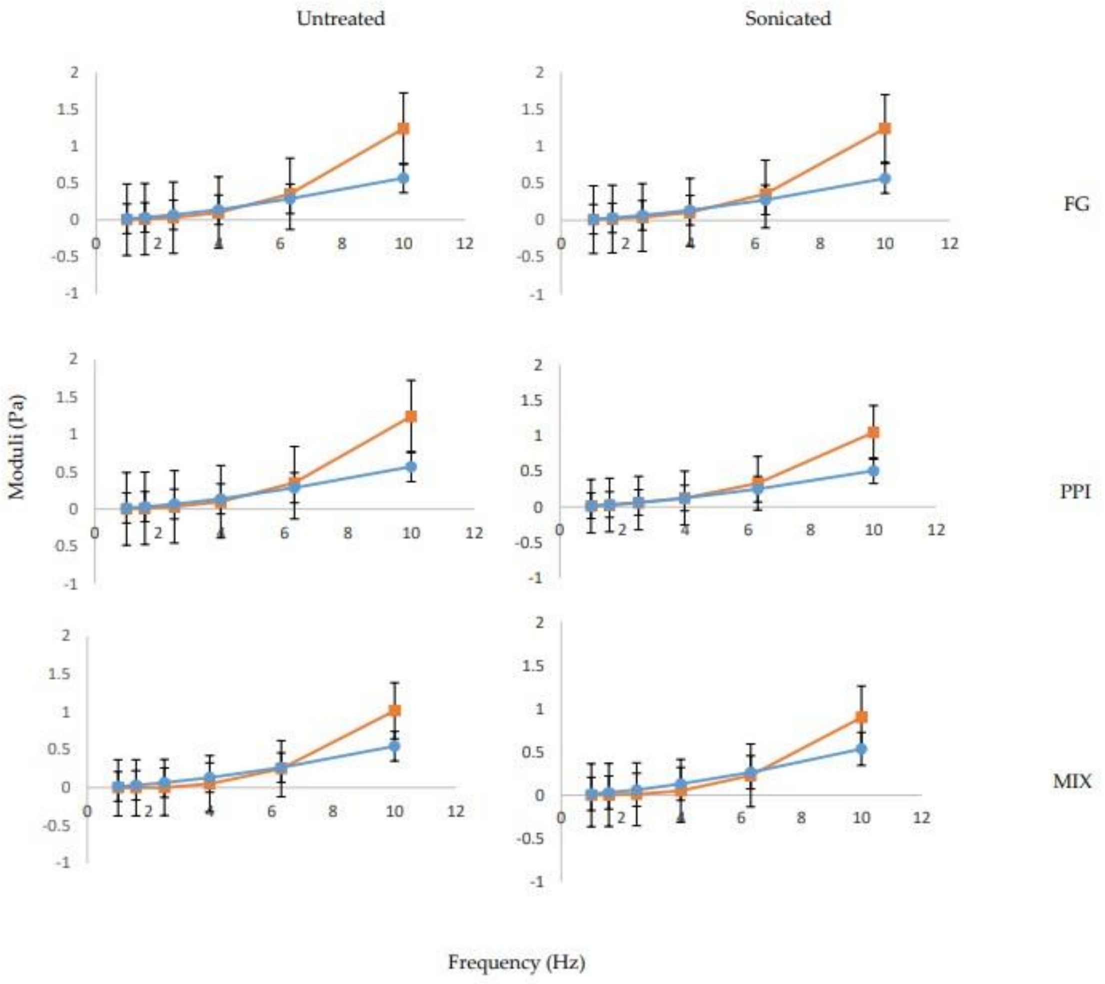

Figure S2 Viscoelastic properties for all the protein solutions at a frequency range between 1-10 Hz. For all the graphs, elastic modulus (G′) is represented in orange squares while viscous modulus (G′′) is represented in blue dots.

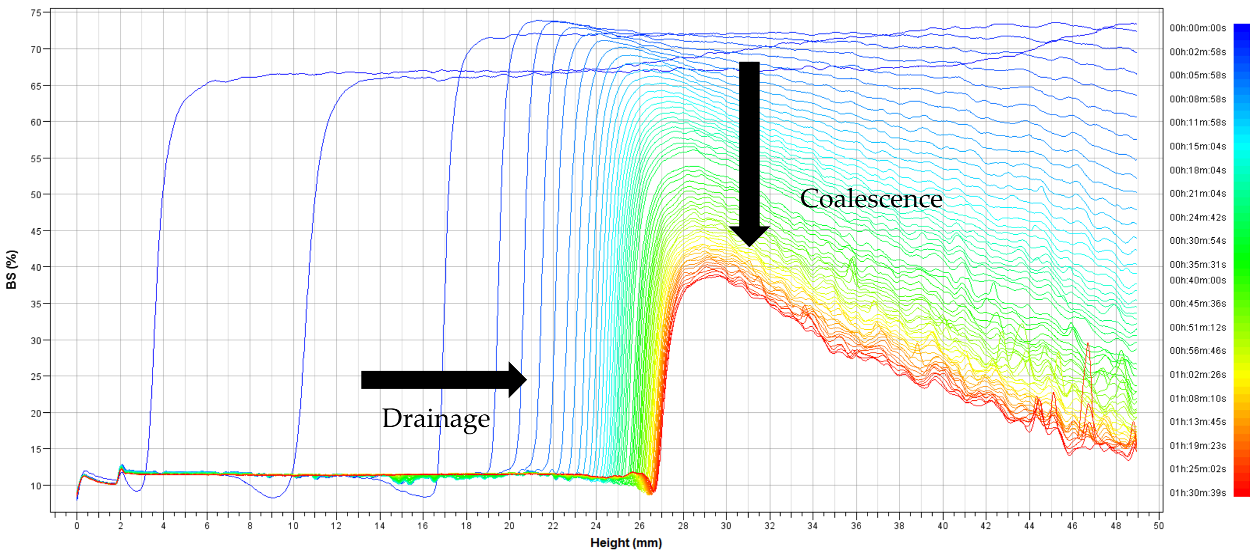

Figure S3. BS values (%) of sonicated PPI over the height of the tube and over time as reported by the instrument Turbiscan Tower. On the right side, scan time is reported with a gradient of colors.
